# Outcome of AIDS-associated cryptococcal meningitis initially treated with 200 mg/day or 400 mg/day of fluconazole

**DOI:** 10.1186/1471-2334-6-118

**Published:** 2006-07-18

**Authors:** CF Schaars, GA Meintjes, C Morroni, FA Post, G Maartens

**Affiliations:** 1 Division of Infectious Diseases, University of Cape Town, Cape Town, South Africa; 2 Division of Clinical Pharmacology, University of Cape Town, Cape Town, South Africa; 3 School of Public Health and Family Medicine, University of Cape Town, Cape Town, South Africa; 4 Canisius Wilhelmina Hospital, Nijmegen, The Netherlands; 5 King's College London School of Medicine, London, UK

## Abstract

**Background:**

AIDS-associated cryptococcal meningitis has a high mortality. Fluconazole was the only systemic antifungal therapy available in our centre. From 1999–2001 we used low-dose fluconazole (200 mg daily initially), and did not offer therapy to patients perceived to have poor prognoses. In 2001 donated fluconazole became available, allowing us to use standard doses (400 mg daily initially). Antiretroviral therapy was not available during the study period.

**Methods:**

Retrospective chart review of adult patients before and after the fluconazole donation.

**Results:**

205 patients fulfilled the inclusion criteria, 77 before and 128 after the donation. Following the donation fewer patients received no antifungal treatment (5% vs 19%, p = 0.002), and more patients received standard-dose fluconazole (90% vs 6%, p < 0.001). In-hospital mortality was 25%. Impaired consciousness, no antifungal treatment received and cerebrospinal fluid antigen titre > 1,000 were independent predictors of in-hospital mortality. Concomitant rifampicin did not affect in-hospital survival. Thirteen patients were referred to the tertiary referral hospital and received initial treatment with amphotericin B for a mean of 6 days – their in-hospital survival was not different from patients who received only fluconazole (p = 0.9). Kaplan-Meier analysis showed no differences in length of survival by initial treatment with standard or low doses of fluconazole (p = 0.27 log rank test); median survival was 76 and 82 days respectively.

**Conclusion:**

Outcome of AIDS-associated cryptococcal meningitis is similar with low or standard doses of fluconazole. The early mortality is high. Initial therapy with amphotericin B and other measures may be needed to improve outcome.

## Background

AIDS-associated cryptococcal meningitis is a severe opportunistic infection with a high mortality, even in developed countries [1]. Early mortality rates in cohorts from the United States have ranged from 11 to 45% [2,3]. Amphotericin B and flucytosine, given for 2 weeks, followed by fluconazole 400 mg daily (reduced to 200 mg daily after 10 weeks) is considered the treatment of choice for AIDS-associated cryptococcal meningitis [4]. The mortality rate with this regimen in a clinical trial was 9.4 % [5]. This low mortality rate is unlikely to be replicated in cohort studies as patients with coma, who have a poorer prognosis, were excluded from the trial.

In developing countries access to antifungal therapy is limited [6]. In sub-Saharan Africa reported mortality rates with antifungal treatment are very high: median survival of 19 days in a Zambian study [7] and 64% in-hospital mortality in a South African study [8]. Factors that are likely to contribute to this high mortality rate include limited access to antifungal therapy, late presentation, inability to adequately monitor intracranial pressure, no use of cotrimoxazole prophylaxis and no access to antiretroviral therapy.

In our centre we had no access to amphotericin B or flucytosine, but had access to fluconazole. Initially, because fluconazole was expensive, we selected patients with fair prognoses and we used low doses: 200 mg daily for initial therapy followed by 100 mg daily for maintenance. There was evidence for this low dose approach: fluconazole 200 mg daily for initial therapy compared to amphotericin B resulted in similar survival and time to negative cultures [9]; and in another trial fluconazole 100 mg daily was used as initial maintenance therapy [10]. Following a fluconazole donation programme by Pfizer Pharmaceuticals [11] we were able to use conventional doses of fluconazole (400 mg daily for initial therapy followed by 200 mg daily for maintenance). In this study we assess the outcome of AIDS-associated cryptococcal meningitis before and during the fluconazole donation programme.

## Methods

A retrospective chart review was conducted at GF Jooste hospital, a public sector secondary level hospital that serves a population of approximately 1.3 million people with a high HIV prevalence. All HIV-infected patients who presented with a first episode of cryptococcal meningitis between January 1999 and December 2002 were included. Donated fluconazole became available in April 2001. Low dose fluconazole (200 mg daily for until CSF was culture negative or for 8 weeks, followed by 100 mg daily for life) was generally used prior to the donation. Furthermore, because of the expense of fluconazole, prior to the donation those patients deemed to have a poor prognosis received no antifungal therapy and were referred for palliative care. The South African protocol for treating cryptococcal meningitis with donated fluconazole was 400 mg daily for 8 weeks followed by 200 mg daily for life. Amphotericin B for initial therapy up to two weeks was optional, but was not available in many smaller hospitals such as ours due largely to intense bed pressure, difficulty in supervising infusions and monitoring for toxicity. Diflucan^®^, Pfizer Inc., was used throughout the study. Amphotericin B (administered at a dose of 0.7 mg/kg/day) was only available at the regional tertiary referral hospital, but following the donation referrals for amphotericin B were no longer accepted. Following discharge most patients were referred to their local primary care clinics for follow up. Antifungal therapy was provided free to patients throughout the study period. During the study period there was no public sector access to antiretroviral therapy.

Patients were identified from computerised laboratory records if one or more of the following tests of cerebrospinal fluid (CSF) was positive: India Ink stain, cryptococcal culture and cryptococcal latex agglutination test titre >1:4 (CLAT, Meridian Bioscience, Cincinatti, OH, USA). Patients were excluded if their HIV status was unknown or negative, or if they received anti-retroviral therapy. Demographic characteristics, prior AIDS (World Health Organisation clinical stage 4), baseline clinical and laboratory data, antifungal treatment, duration of hospitalisation and outcome were extracted from the medical records. The level of consciousness at the time of admission was classified into three stages using a modified version of the classification used for tuberculous meningitis [12]: stage 1 fully conscious (Glasgow Coma Scale [GCS] 15/15) without neurological deficit, stage 2 focal neurological signs such as squint or hemiparesis and/or GCS 10–14, and stage 3 flaccid hemi- or paraplegia and/or GCS below 10/15. Entries made in the medical records were interpreted by the first author and when ambiguous by the first two authors.

Data were analyzed using Stata 8.0 (Stata Corporation, College Station, Texas, USA). Bivariate associations were described using chi-square, Fisher's exact and Wilcoxon rank-sum tests as appropriate. Because we were unable to ascertain date of death for patients referred to palliative care facilities, we analysed survival as the time to the combined endpoint of date of death or transfer to the palliative care facility. Kaplan-Meier survival curves were generated and survival expressed in days from the date of diagnosis to the time of death or transfer to a palliative care facility (failures) or the date of the last visit when the patient was known to be well (censored). Survival curves were compared using the log-rank test for the equality of the survivor function across groups.

The study was approved by the Research Ethics Committee, University of Cape Town.

## Results

A total of 249 patients with cryptococcal meningitis were identified during the study period: 44 were excluded from the study (9 missing charts, 19 HIV status not recorded, 5 HIV seronegative, 5 relapsed cryptococcal meningitis, 3 on antiretroviral therapy, 3 CLAT titre ≤1:4) leaving 205 evaluated patients. Demographic, laboratory, clinical and antifungal treatment data of the patients before and during the donation programme are shown in Table 1. Most patients were black Africans. Cryptococcal meningitis was the initial WHO stage 4 condition in 78% of patients. Sixty-nine percent of patients had prior tuberculosis (extrapulmonary in 19%), while 41% were receiving anti-tuberculous treatment at the time of presentation.

**Table 1:** Clinical and laboratory parameters of patients diagnosed as having AIDS-associated cryptococcal meningitis between January 1999-March 2001 and from April 2001-December 2002 (before and during the fluconazole donation programme respectively)

	Before April 2001 (n = 77)	From April 2001 (n = 128)	P value
Median age in years (range)	33 (17–75)	35 (21–66)	0.4
Male sex	36 (47%)	63 (49%)	0.7
Prior AIDS-defining illness	20 (26%)	26 (20%)	0.3
Prior tuberculosis	51 (66%)	91 (71%)	0.5
Concurrent TB treatment	33 (43%)	52 (41%)	0.8
CSF India Ink	(n = 68)	(n = 121)	0.2
Positive	56 (82%)	88 (73%)	0.2
CSF CLAT titer	(n = 50)	(n = 82)	0.9
≥ 1000	36 (72%)	57 (70%)	0.9
< 1000	14 (28%)	25 (30%)	
CSF fungal culture	(n = 29)	(n = 66)	0.07
Positive	27 (93%)	51 (77%)	0.1
Level of consciousness			
Stage 1	45 (58%)	70 (55%)	0.9
Stage 2	30 (39%)	54 (42%)	
Stage 3	2 (3%)	4 (3%)	
Therapeutic lumbar punctures	(n = 7)	(n = 23)	0.12
Single	4	14	1.0
Multiple	3	9	
Any treatment received			
Yes	62 (81%)	121 (95%)	<0.01
No	15 (19%)	7 (5%)	
Type of initial treatment received	(n = 62)	(n = 121)	
Fluconazole 200	45 (73%)	12 (10%)	<0.001
Fluconazole 400	4 (6%)	109 (90%)	
Amphotericin B*	13 (21%)	0 (0%)	

*This was only available at the local tertiary referral hospital. Following the donation referrals for amphotericin B were no longer accepted.

CSF pleocytosis (>3 cells/μL) was present in 69% of patients, with lymphocytic predominance in 82%. Median CSF leukocyte count was 16 cells/μL. CSF protein was elevated (>0.4 g/L) in 92% and hypoglycorrhachia (CSF glucose <2.2 mmol/L) was present in 59%. In total 12 (6%) patients had a normal CSF (no pleiocytosis, protein ≤0.40 g/L and glucose ≥2.2 mmol/L). CSF pressure was not recorded due to the absence of manometers. The median CD4^+ ^T-lymphocyte count was 34 cells/μL (range 3–186), but was only performed in 30 patients.

No significant baseline differences were noted between patients who received cryptococcal meningitis treatment prior to or during the fluconazole donation programme (Table 1). However, when fluconazole became available through the donation programme there were significant differences in the antifungal treatment received: more patients received higher dose fluconazole, no patients received initial amphotericin B therapy (this was due to a policy change at the tertiary hospital) and fewer patients received no antifungal treatment (Table 1). In total 22 patients received no antifungal treatment: 6 died before treatment (4 in the pre-donation period); 6 patients received only palliative care (all in the pre-donation period); 2 did not receive treatment because of health system shortages; 5 patients were mis-diagnosed as tuberculous meningitis (2 post-donation); and 3 patients (all post-donation) did not report for their results of the CSF examination.

The median duration of the first hospital stay was 5 days. Fifty-two patients (25%) died during the first hospital admission after a median of 6 days (range 1–97 days), and 4 patients (2%) were discharged to a palliative care facility. Of the 149 patients who left the hospital alive and were not discharged for palliative care, 110 (74%) were discharged without disability, 27 (18%) had neurological impairment, and in 12 (8%) the neurological status upon discharge was not recorded. There were no significant differences in length of hospital stay, inpatient mortality or outcome in the pre- compared with the post-donation period (Table 2).

**Table 2:** Inpatient outcomes for the first hospital admission between January 1999 and March 2001 (pre-donation period) and between April 2001 and December 2002

Outcome	Before April 2001 Pre-donation n = 77	After April 2001 Donation n = 128	P-value for comparison	Overall n = 205
Length of hospital stay, median days (IQR)				
GF Jooste hospital	4 (2.5–9)	5 (3–9)	0.2	5 (3–9)
Tertiary hospital	7 (4–12.5)	4	0.4	7 (4–11.8)
Total hospital stay	6 (4–11)	5 (3–9)	0.4	5 (3–10)
				
In hospital mortality, n (%)	18 (23.4)	34 (26.6)	0.6	52 (25.4)
				
Discharge outcome, n (%)				
No disability	44 (57.1)	66 (51.6)	0.8	110 (53.7)
Disability	11 (14.3)	16 (12.5)		27 (13.2)
Unspecified disability	3 (3.9)	9 (7)		12 (5.9)
Palliative care	1 (1.3)	3 (2.3)		56 (27.3)

In univariate analysis in-hospital mortality was not significantly different between patients who received initial fluconazole 400 mg daily versus fluconazole 200 mg daily initially (p = 0.4) or between patients who received initial amphotericin B and fluconazole at any dose (p = 0.9). Patients who received no antifungal treatment, those with lower level of consciousness, and CSF CLAT titre > 1,000 had a higher risk of the combined endpoint of in-hospital death or palliative care facility transfer in the Cox proportional hazard model (Table 3). Patients with Stage 3 level of consciousness had 100% in-hospital mortality, compared with 43% for those with Stage 2, and 12% for those with Stage 1. Concomitant anti-tuberculous therapy including rifampicin, which reduces fluconazole levels by induction of cytochrome P450 enzymes [13], was not associated with in-hospital death or palliative care facility transfer.

**Table 3:** Cox proportional hazard model of time to the combined endpoint of inpatient death or transfer to palliative care facility for the first hospital admission.

**Variable**	**Hazard ratio (95% confidence interval)**
Initial treatment	
No treatment	1 (referent)
Amphotericin B	0.23 (0.09–0.61)
Fluconazole 200	0.34 (0.17–0.68)
Fluconazole 400	0.38 (0.21–0.68)

Level of consciousness	
Stage 1	1 (referent)
Stage 2	2.51 (1.65–3.8)
Stage 3	5.95 (2.37–14.94)

CSF CLAT titre	
≤ 1,000	1 (referent)
> 1,000	1.74 (1.01–3.01)

Concomitant rifampicin	1.21 (0.81–1.8)

**Figure 1:**
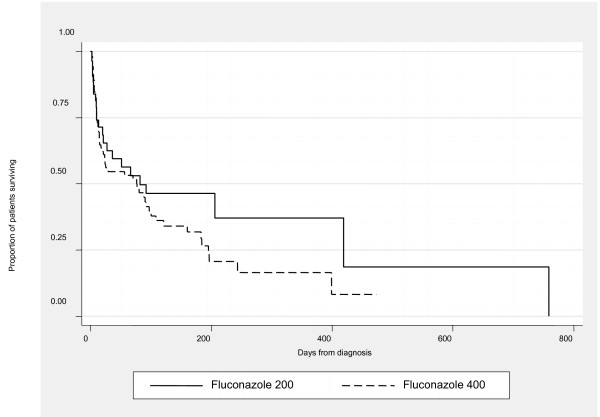
Kaplan-Meier survival curves for AIDS-associated cryptococcal meningitis treated with low (200 mg daily) and standard (400 mg daily) dose fluconazole (p = 0.27, log rank test).

Median follow up for the patients discharged from hospital alive was 36 days. The median survival from time of cryptococcal meningitis diagnosis was 76 days; 10% of patients were known to be alive after six months and 3% after one year. Kaplan-Meier analysis showed no overall differences in length of survival by initial treatment with fluconazole 400 mg versus fluconazole 200 mg (p = 0.27 log rank test) (Figure 1); median survival was 76 and 82 days respectively.

## Discussion

The fluconazole donation has enabled us to treat all patients with AIDS-associated cryptococcal meningitis with antifungal treatment. In addition, it enabled us to use standard-dose fluconazole. However, despite similar baseline characteristics, mortality was not significantly improved by the fluconazole donation. There may have been other benefits that were not measured in this study. Firstly, the savings to the pharmaceutical budget were not calculated. We have previously shown that the net treatment costs of cryptococcal meningitis were $ 2,620, of which 26% were pharmacy and clinic visit costs [14]. Secondly, the impact on staff morale was not assessed – denying patients effective therapy, as was done in the pre-donation period, is stressful for healthcare professionals. Thirdly, the fluconazole donation has also allowed appropriate treatment for oesophageal candidiasis, which has a major impact on the quality of life of HIV-infected patients. The impact in most other sub-Saharan African countries, where access to antifungal treatment was extremely limited [7], has presumably been greater than we observed.

Although our mortality rates are high, they are similar to those reported from two cohorts from the United States [2,3]. However the treatment for cryptococcal meningitis was not specified in the one study [2] and doses of Amphotericin B varied in the other [3]. Thus it is unclear what the outcome would be with treatment regarded as the current standard of care [4]. The mortality we observed was lower than in the two other African studies where antifungal treatment was used under standard conditions (i.e. not in clinical trials). In Zambia [7] the median survival for those without antifungal treatment was 10 days, increasing only to 19 days with fluconazole treatment (400 mg immediate dose followed by 200 mg daily), leading the authors to conclude "patients receiving fluconazole did not show a significant survival advantage". In a South African study [8] the in-hospital mortality with antifungal treatment (amphotericin B, sometimes with flucytosine, or fluconazole – but there was no analysis of outcomes by treatment group) was 64%. The reasons for the better survival rates in our study compared with the other African studies are unclear. Cryptococcal meningitis was the initial AIDS-defining illness in 78% of our patients, which is comparable to the 84% found in the other South African study [8] and the 91% found in Zambia [7]. It is not possible to directly compare level of consciousness on admission, as a marker of late presentation, as the other studies did not report this in the same manner we did. Nevertheless, the fact that 13% in the Zambian study had confusion [7] and 36% in the other South African study had abnormal mental status [8] compared to 44% with grades 2 or 3 level of consciousness in our study, suggests that this is not the explanation. Therapeutic lumbar punctures, which are thought to improve outcome [4], were only performed in 15% of patients in our study, but were not reported in the other two African studies. We routinely used prophylactic cotrimoxazole throughout the study period, which was not used in Africa when the other two studies were done. However, although cotrimoxazole reduces mortality in South African patients with AIDS [15], it is unlikely that it would have a dramatic impact on early mortality.

None of the patients in this study were receiving antiretroviral therapy, which is now becoming available in sub-Saharan Africa and other developing countries on a wide scale. Antiretroviral therapy will undoubtedly improve the prognosis of patients surviving their hospital stay, but is unlikely to impact on the high in-patient mortality.

Tuberculosis is highly prevalent in HIV-infected patients in South Africa, as illustrated by the finding that 41% in our series and 32% in another South African study [8] were receiving treatment for tuberculosis at the time of presentation. Cryptococcal meningitis can be misdiagnosed as tuberculous meningitis, as occurred in 5 patients in our series and in 2 of 44 patients in another South African study [8]. Rifampicin is a potent inducer of the cytochrome P450 enzyme system, of which fluconazole is a substrate. Concomitant rifampicin resulted in a 22% reduction of the fluconazole area under the concentration-time curve in a Thai study of AIDS-associated cryptococcal meningitis [13], but there were no differences in the rate of CSF cryptococcal culture conversion in the rifampicin group. However, the authors expressed concern that patients receiving rifampicin whilst on the fluconazole 200 mg daily maintenance dose had fluconazole concentrations that were below the usual *C. neoformans *minimal inhibitory concentration for much of the time [13]. In our study concomitant rifampicin did not increase the risk of in-hospital mortality, even in patients receiving initial therapy with fluconazole 200 mg daily.

There was no difference in outcome between the low-dose (initial therapy 200 mg daily and maintenance therapy 100 mg daily) and standard-dose (initial therapy 400 mg daily and maintenance therapy 200 mg daily) fluconazole groups. We have previously reported 7-month median survival in patients surviving until hospital discharge using the low-dose fluconazole strategy in a cohort followed up at hospital-based HIV clinics [16]. However we would not advocate the use of low-dose fluconazole as this could select for resistance, particularly as we are experiencing increasing MICs to fluconazole, as has been reported in Cambodia [17].

This study has several limitations. Firstly, it is a retrospective study and comparisons between different treatment groups should be made with caution. Secondly, as noted in the discussion on initial treatment with amphotericin B, some of the groups were too small to draw meaningful conclusions. Thirdly, follow-up after hospital discharge was poor. Our centre was operating largely as an acute care hospital with very limited outpatient facilities during the study period. However, most of the analyses were done on hospital outcomes.

## Conclusion

The fluconazole donation has allowed us access to standard-dose fluconazole treatment for AIDS-associated cryptococcal meningitis. The outcome appeared no better than when we were selecting patients for low-dose fluconazole therapy, but other benefits of the fluconazole donation were not measured. The early mortality in our study was better than in other African studies. Fluconazole monotherapy may be an option in resource-poor settings with limited access to antifungal therapy.

## Competing interests

The author(s) declare that they have no competing interests.

## Authors' contributions

CFS drafted the manuscript and acquired the data. GAM and FAP helped to draft the manuscript and contributed to study design. CM performed the statistical analyses and helped to draft the manuscript. GM conceived the study, participated in study design, and helped to draft the manuscript. All authors read and approved the final manuscript.

## Pre-publication history

The pre-publication history for this paper can be accessed here:


